# Flash Communication: Sulfonyl Fluoride Activation via S–F and C–S Bond Cleavage by a Ni(0) Bis-Bidentate *N*‑Heterocyclic Carbene Complex

**DOI:** 10.1021/acs.organomet.5c00144

**Published:** 2025-07-10

**Authors:** Ethan B. Chavarin, Zoe Y. Marr, Jacob P. Brannon, Andressa Antonini Bertolazzo, Gregory A. Barding, Nicholas Ball, S. Chantal E. Stieber

**Affiliations:** † Chemistry & Biochemistry Department, 6647 California State Polytechnic University, Pomona, California 91768, United States; ‡ Department of Chemistry & Biochemistry, Weber State University, Ogden, Utah 84408, United States; § Department of Chemistry, 2529 Pomona College, Claremont, California 91711, United States

## Abstract

This work investigates possible mechanisms and intermediates in the reactivity of *p*-toluenesulfonyl fluoride with a new bidentate *N*-heterocyclic carbene (NHC) nickel complex, (^Mes^NHC_2_^o^Xy)­Ni­(COD) (Mes = 2,4,6-trimethylphenyl, ^o^Xy = *ortho*-xylyl, COD = cyclooctadiene). (^Mes^NHC_2_^o^Xy)­Ni­(COD) was synthesized from the corresponding bis­(imidazolium) salt precursor, [^Mes^NHC_2_^o^Xy]­[Br]_2_, and both were structurally characterized. (^Mes^NHC_2_^o^Xy)­Ni­(COD) reacts with 1 equiv of *p*-toluenesulfonyl fluoride to furnish (^Mes^NHC_2_^o^Xy)­Ni­(η^2^-SO_2_), HF, and 1/2 equiv of 4,4′-dimethylbiphenyl. (^Mes^NHC_2_^o^Xy)­Ni­(η^2^-SO_2_) was structurally characterized and has a unique side-on SO_2_ coordination mode with a Ni–S–O angle of 60.49(5)°. DFT calculations of (^Mes^NHC_2_^o^Xy)­Ni­(η^2^-SO_2_) are consistent with a Ni­(II) center and an activated SO_2_ fragment. DFT calculations also support an initial oxidative addition at either the S–F or S–C position, which have similar energetics.

## Graphical abstract

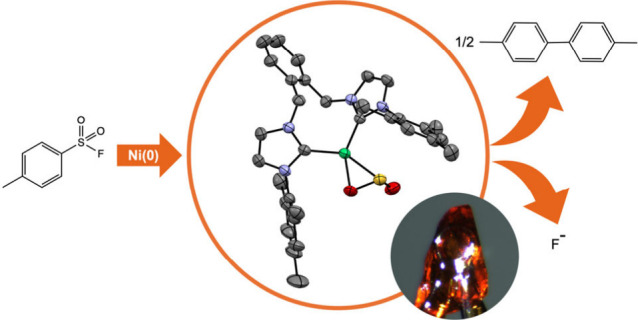

Sulfonyl fluorides are of particular interest as synthons in sulfur­(IV) fluoride exchange (SuFEx) reactions,^1–3^ biological applications,^4^ materials chemistry, chemical cross-linking mass spectrometry,^5^ and drug discovery. Sulfur­(VI)-fluorides can be activated vis SuFEx or C–S/S-F bond cleavage,^6^ and are widely used in sulfur­(VI)-fluoride exchange reactions as “click” reagents.^3,6^ Alternative approaches to activate sulfonyl fluorides have primarily involved Pd, Ni, and Cu catalysts toward C–C bond formation.^7^

The reactivity of sulfonyl fluorides with transition metals is an area that is of growing interest for possible applications of sulfonyl fluorides in catalysis.^8^ Specific questions remain regarding the nature of the C–S bond oxidative addition and the potential for competing S–F bond activation.

Known examples of S–F bond activation feature inorganic S­(VI) fluorides.^9,10^ A Pt(0) NHC complex facilitated an oxidative addition and S–F activation when SOF_2_ was added, resulting in a *trans*-addition of one fluoride and Pt-(S=O)F coordination.^9^ Another study demonstrated the activation of a Pt-SF_3_ complex with ethanol toward a Pt-(S=O)F complex featuring phosphine ligands.^10^ Prior studies with metal powders or oxides of Ag, Zn, U, and Cu resulted in either cleavage of all S–F and S=O bonds, or metal fluorination with release of SO_2_.^11,12^ There is less direct evidence of activation of organic sulfur fluorides—especially organosulfur­(VI) fluorides—despite their emerging use in catalytic desulfonative reactions.^13,14^ Considering the numerous examples of C–F^15,15–19^ and S–C^20–23^ bond activation, as well as decarbonylative couplings^24^ using Ni catalysts, we hypothesize Ni-complexes could serve as a model for investigating modalites of activating organic S­(VI) fluorides. Desulfonylative Suzuki-Miyaura reactions using sulfonyl fluorides generally feature Pd-catalysts, with transmetalation hypothesized to be the rate-determining step.^25^ A recent report of nickel catalysts for desulfonylative cross-coupling of aryl sulfones with aryl bromides primarily used *Ar*-SO_2_–CF_3_ as a substrate, however phenyl sulfonyl fluoride was reported in a control experiment to yield some cross-coupled product.^13^ This suggests that Ni can directly activate aryl sulfonyl fluorides. Understanding how Ni can activate sulfonyl fluorides is important in developing cheaper and more earth-abundant metal catalyzed reactions using sulfonyl fluorides. This work reports a bidentate NHC Ni(0) complex that activates *p*-toluenesulfonyl fluoride by breaking the C–S and S–F bonds to form a Ni-SO_2_ complex with a new side-on SO_2_ coordination mode.

Bis-monodentate NHC nickel(0) complexes have been reported for C–F activation of aryl fluorides, however they result in *trans*-oxidative addition of the resulting aryl fluoride,^15,16^ potentially limiting further reactivity. In the current work, bis-bidentate NHC nickel complexes were chosen with the intention that the chelating ligand could promote oxidative addition and reductive elimination mechanisms that necessitate *cis*-substrate orientations.^26^ The ligand precursor [^Mes^NHC_2_^o^Xy]­[Br]_2_ (Mes = 2,4,6-trimethylphenyl, ^o^Xy = *ortho*-xylyl) **1**, was synthesized as previously reported from 2 equiv of mesityl imidazole with 1,2-bis­(bromomethyl)­benzene, and was structurally characterized (Figure 1).^27^ [^Mes^NHC_2_^o^Xy]­[Br]_2_ **1** has mesityl wingtips and an *ortho*-xylyl linker, which provides flexibility in overall ligand backbone. Deprotonation with 2 equiv of potassium bis­(trimethylsilyl)­amide (KHMDS) followed by addition of Ni­(COD)_2_ (COD = cyclooctadiene), resulted in isolation of a new Ni(0) complex, (^Mes^NHC_2_^o^Xy)­Ni­(COD) **2**, which was structurally characterized (Figure 2). The COD ligand is coordinated in an η^2^ fashion, in contrast to our previously reported related complex with a methylene linker that has a κ^2^,η^2^-coordinated COD ligand, (^Mes^NHC_2_Me)­Ni­(COD).^28^

**Figure 1:**
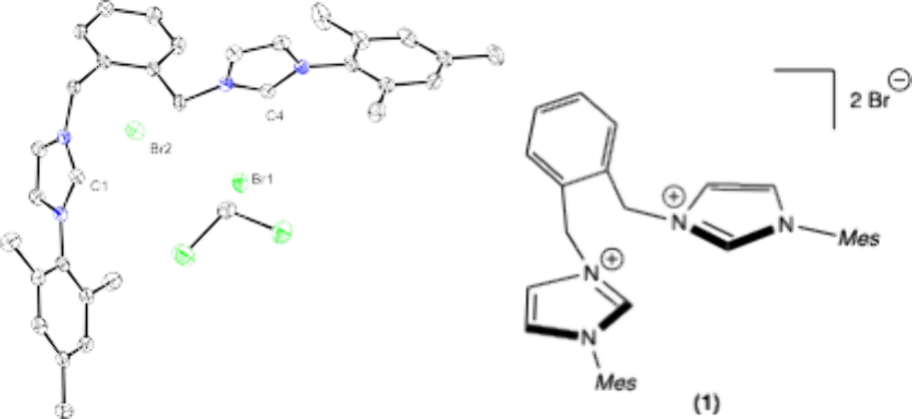
Solid state structure of [^Mes^NHC_2_^o^Xy]­[Br]_2_ **1** at 50% probability ellipsoids (left) and drawing of structure (right), with H atoms removed for clarity.

**Figure 2:**
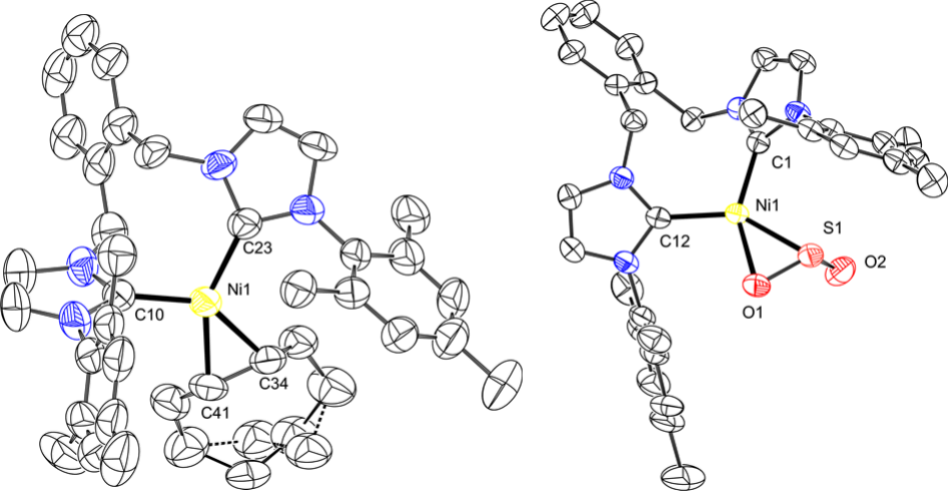
Solid state structures of (^Mes^NHC_2_^o^Xy)­Ni­(COD) **2** at 50% probability ellipsoids (left) and (^Mes^NHC_2_^o^Xy)­Ni­(η^2^-SO_2_) **3** (right), with H atoms and solvent removed for clarity.

A stoichiometric reaction of (^Mes^NHC_2_^o^Xy)­Ni­(COD) **2** with *p*-toluenesulfonyl fluoride resulted in formation of a Ni-SO_2_ complex, (^Mes^NHC_2_^o^Xy)­Ni­(η^2^-SO_2_) **3** with 68% yield, which was characterized by ^1^H and ^13^C NMR, FTIR, and X-ray crystallography (Scheme 1, Figure 2). The Ni-SO_2_ FTIR stretches are assigned at 1040 cm^–1^ and 848 cm^–1^ (see Supporting Information), which are shifted from the SO_2_ stretches in *p*-toluenesulfonyl fluoride at 1401 cm^–1^ and 1202 cm^–1^. These are consistent with reported IR stretching frequencies for V, Rh, and Ir-SO_2_ complexes.^29,30^ Initially it was assumed that *p*-fluorotoluene would be the associated byproduct of this reaction, however *p*-fluorotoluene was not detected by ^19^F NMR or GC-MS. Instead, after an NMR tube reaction, the organic byproduct 4,4′-dimethylbiphenyl was detected by GC-MS and HF was observed in the ^1^H NMR (see Supporting Information). It should be noted that HF formation presents a significant chemical hazard. The proton source for HF formation likely was from the THF solvent or a result of advantitious water. Attempts to intercept the fluoride with silyl reagents or CsCl were unsuccessful. An NMR-tube reaction with with arrayed spectra over the course of 1 h after adding CsCl had ^19^F signals at −31 ppm and −227 ppm, which are attributed to SO_2_F_2_ and F^–^, respectively (see Supporting Information).^31,32^ A homocoupled product was also reported in the desulfonylative cross-coupling of trifluoromethyl aryl sulfones with aryl bromides,^13^ indicating a potential common mechanistic pathway for sulfones.

**Scheme 1:**
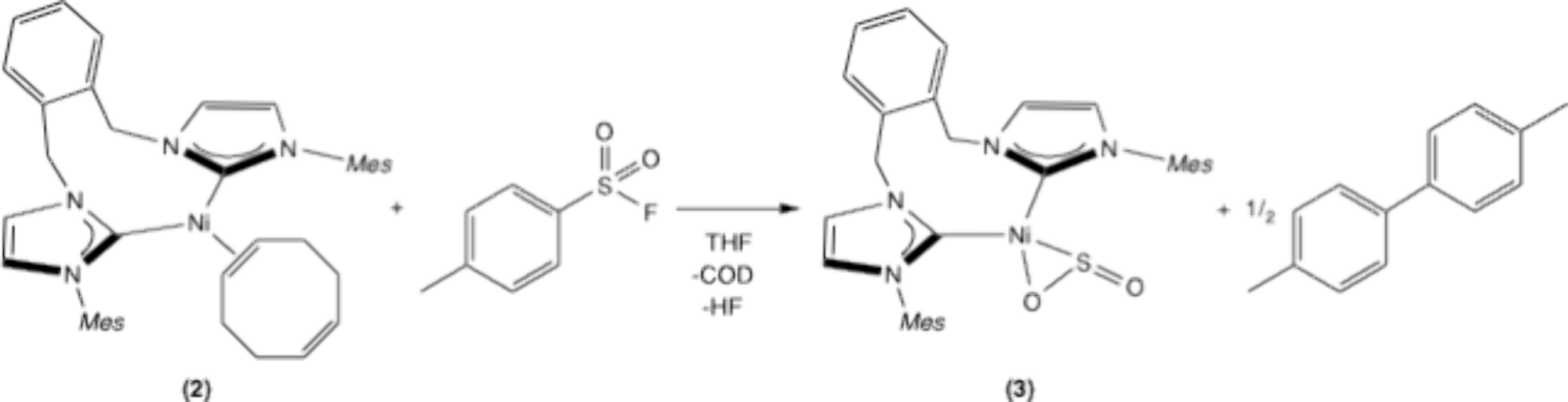
Reactivity of (^Mes^NHC_2_^o^Xy)­Ni­(COD) **2** with p-Toluenesulfonyl Fluoride to Form (^Mes^NHC_2_^o^Xy)­Ni­(η^2^-SO_2_) **3**

To the best of our knowledge, there are only two other reported Ni-SO_2_ complexes, which were both were synthesized through the addition of SO_2_ gas.^33,34^ The reported structures display predominantly Ni–S coordination as represented by the Ni1–S1–O1 and Ni1–S1–O2 angles in the range of 106–126°. Surprisingly, our new (^Mes^NHC_2_^o^Xy)­Ni­(η^2^-SO_2_) complex **3** displays a “side-on” η^2^-SO_2_ coordination with a Ni1–S1–O1 angle of 60.49(5)° and a Ni1–S1–O2 angle of 113.12(7)°. Previous computational work on η^1^-SO_2_ versus η^2^-SO_2_ coordination in pentacarbonyl complexes suggested that η^2^-SO_2_ coordination is favored for more electron rich metals and that SO_2_ should be thought of as a weak σ-donor with strong π-backbonding.^35^ These three structural examples of Ni-SO_2_ complexes indicate that the denticity of the ancillary ligand may also be a factor, with tri- and tetradentate ancillary ligands supporting η^1^-SO_2_ coordination,^33,34^ and a bidentate ancillary ligand supporting η^2^-SO_2_ coordination.

The SO_2_ fragment in (^Mes^NHC_2_^o^Xy)­Ni­(η^2^-SO_2_) is relatively activated, with an S1–O1 bond distance of 1.5428(15) Å. By comparison, the S–O bond distances are 1.430(15) Å in gaseous SO_2_,^36^ and 1.2128–1.450 Å in the previously reported Ni-SO_2_ complexes.^33,34^ The second S–O bond distance in (^Mes^NHC_2_^o^Xy)­Ni­(η^2^-SO_2_) is 1.4848(16) Å for S1–O2, and is also slightly longer than the S–O distances in the previously reported Ni-SO_2_ complexes.^33,34^ Combined, the structural data support an activated SO_2_ fragment as compared to gaseous SO_2_.

A geometry optimization calculation using density functional theory was conducted of (^Mes^NHC_2_^o^Xy)­Ni­(η^2^-SO_2_) **3** using the ORCA program^37,38^ to gain insights toward the unique Ni1–S1–O1 bond angle and resulting electronic structure. The open-shell calculation (UKS) used the full molecule, B3LYP functional, RIJCOSX approximation, the def2-TZVP­(-f)/J basis set on Ni and atoms coordinated to the metal, and the def2-SVP/J basis set for all other atoms. Both an *S* = 0 and an *S* = 1 input were probed. The *S* = 0 input resulted in bond distances and angles within experimental error (SI), while the *S* = 1 input resulted in distortion of the Ni–S–O angle and could not be converged. The resulting qualitative *d*-orbital splitting diagram from the *S* = 0 calculation is consistent with a Ni­(II) center (Figure 3) with an unoccupied *d*_*x* ^2^–*y* ^2^_ orbital. Both the HOMO and LUMO are ligand-based on SO_2_ and the xylyl linker, respectively. The calculated bond distances of 1.559 Å for S1–O1 and 1.492 Å for S1–O2 are within reasonable agreement of the experimental distances of 1.5428(15) Å for S1–O1 and 1.4848(16) Å for S1–O2. A frequency calculation at the BP86 level of theory resulted in a calculated asymmetric SO_2_ stretch at 1075 cm^–1^ and a symmetric SO_2_ stretch at 841 cm^–1^, which are in agreement with the experimental values of 1040 cm^–1^ and 848 cm^–1^, respectively. Combined with the geometry optimization of the (^Mes^NHC_2_^o^Xy)­Ni­(COD) **2** starting material, the calculations support oxidation from Ni^0^ to Ni^II^ and with a side-on activated SO_2_.

**Figure 3:**
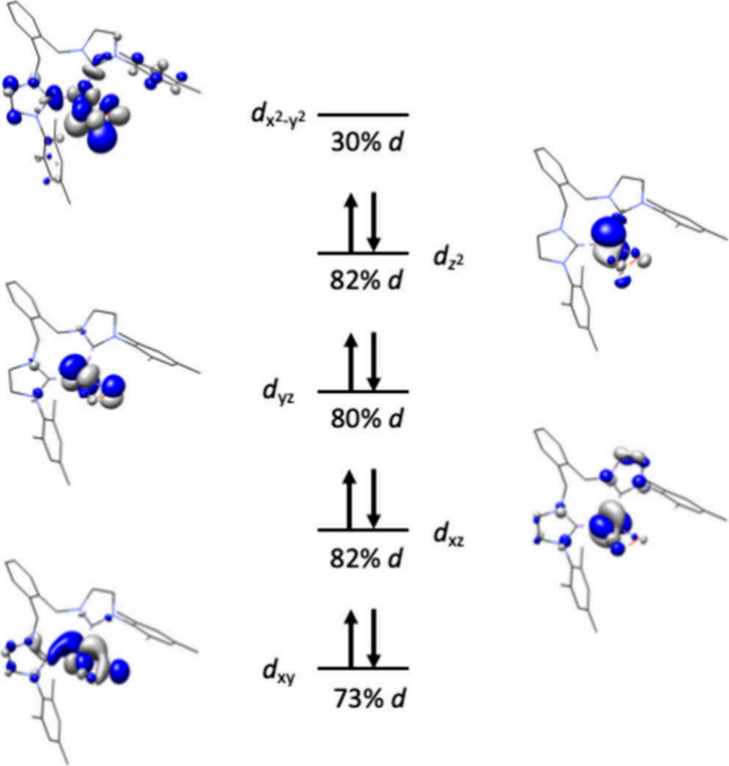
Qualitative molecular orbital diagram of (^Mes^NHC_2_^o^Xy)­Ni­(η^2^-SO_2_) **3** resulting from B3LYP geometry optimization.

It is particularly interesting that Pd systems and one Ni system are reported to catalyze formation of sulfonyl fluorides,^39–41^ while (^Mes^NHC_2_^o^Xy)­Ni­(COD) **2** facilitates the reverse reaction. To probe this further, computational studies examined the possibility of C–S versus S–F bond activation. The first step is proposed to be COD ligand dissociation, followed by oxidative addition of *p*-toluenesulfonyl fluoride. Structures of the proposed C–S and S–F oxidative addition products were built and roughly optimized in Avogadro, followed by full optimization with the ORCA program.^37^ The S–C activated product was calculated at 5.5 kcal/mol lower energy than the S–F activated product, although both are energetically reasonable. This is similar to mechanistic studies with palladium, where S–C bond oxidative addition is proposed to be the first step.^42^ One possibility, is that after oxidative addition of the S–C bond, there is a transmetalation step between two nickel centers to form a bis­(*p*-toluene) Ni­(II) that can readily undergo reductive elimination to form 4,4′-dimethylbiphenyl. Complex **3** could then be formed by a 1,2-elimination of SO_2_F_2_ from a bis­(SO_2_F) Ni complex. A full mechanistic study is underway.

In this study, S–F and C–S activation of *p*-toluenesulfonyl fluoride is reported with a novel Ni(0) complex, (^Mes^NHC_2_^o^Xy)­Ni­(COD) **2**, to form a product with SO_2_ coordinated to Ni. The resulting complex (^Mes^NHC_2_^o^Xy)­Ni­(η^2^-SO_2_) **3** is unique in having a side-on coordinated SO_2_, which was structurally characterized and has not been previously reported for nickel. The Ni­(II) oxidation state with an activated SO_2_ moiety, offers the potential for further SO_2_ functionalization. A proposed reaction pathway involves oxidative addition of *p*-toluenesulfonyl fluoride via S–F or C–S activation, and both pathways were found to be reasonable based on DFT calculations. The formation of 4,4′-dimethylbiphenyl is the homocoupled product which was formed along with 1 equiv of HF. These results offer insights toward the reactivity of nickel with sulfonyl fluorides and highlights the potential for these systems in both cross-coupling and SO_2_ activation with impacts for SuFEx and pharmaceutical chemistry. Future studies will be carried out in our laboratories to evaluate the mechanisms and reactivity of these complexes.

## Supplementary Material



## Data Availability

Zenodo contains supplementary experimental and computational data files (.fid, .mnova., .txt., .spc., .inp, .out, .xyz) under DOI: 10.5281/zenodo.15083911. These data can be obtained free of charge via https://zenodo.org.

## Notes

Experimental data, spectral data, and computational summary (PDF)

## Abbreviations

NHC: *N*-heterocyclic carbene
KHMDS: potassium bis­(trimethylsilyl)­amide.
