# Supplementary material for: Flash Communication: Sulfonyl Fluoride Activation via S–F and C–S Bond Cleavage by a Ni(0) Bis-Bidentate N‑Heterocyclic Carbene Complex
Source: Organometallics. 2025 Jul 10;44(17):1865–9. doi: 10.1021/acs.organomet.5c00144 (PMC12421660; doi:10.1021/acs.organomet.5c00144)
Supplement: Supplementary file 1 [file om5c00144_si_001.pdf]

Supporting Information

Flash Communication: Sulfonyl fluoride activation via S-F and C-S bond cleavage by a Ni(0) bis-bidentate *N*-heterocyclic carbene complex

*Ethan B. Chavarin,[1] Zoe Y. Marr,[1] Jacob P. Brannon,[1] Andressa Antonini Bertolazzo,[1,2] Gregory A. Barding,[1] Nicholas Ball,[3] S. Chantal E. Stieber[1]**

[1]Chemistry & Biochemistry Department, California State Polytechnic University, Pomona, California 91768, United States
[2]Department of Chemistry & Biochemistry, Weber State University, Ogden, Utah 84408, United States.
[3]Department of Chemistry, Pomona College, Claremont, California 91711, United States

sestieber@cpp.edu

Table of Contents

1. Procedures, Materials, and Instrumentation S3

   1.1 General Considerations S3

   1.2 Instrumentation and Software S3

   1.3 Abbreviations S5

2. Synthetic Procedures S5

   2.1 Synthesis of [^{Mes}NHC$_2$^{o}Xy][Br]$_2$ S5

   2.2 Synthesis of (^{Mes}NHC$_2$^{o}Xy)Ni(COD) S6

   2.3 Synthesis of (^{Mes}NHC$_2$^{o}Xy)Ni(SO$_2$) S10

3. Additional characterization, including NMR, IR, GC-MS S14

4. Crystallographic Data S20

   4.1 [^{Mes}NHC$_2$^{o}Xy][Br]$_2$ S20

   2.1 (^{Mes}NHC$_2$^{o}Xy)Ni(COD) S21

   2.2 (^{Mes}NHC$_2$^{o}Xy)Ni(SO$_2$) S23

5. Computational Analysis S25

6. References S26

1. Procedures, Materials, and Instrumentation

1.1 General Considerations

All air- and moisture-sensitive manipulations were carried out using in an MBraun inert atmosphere (nitrogen) dry box, unless otherwise noted. All glassware was stored at 150 °C in a pre-heated oven prior to transferring to the glovebox. Solid reagents were dried overnight on a vacuum line with a needle valve, prior to transferring to the glovebox. The solvents used for air- and moisture-sensitive manipulations were dried and deoxygenated with a JC Meyer solvent purification system and transferred to the glovebox using a Strauss flask. All reagents were purchased from commercial suppliers and used without further purification unless otherwise noted. Mesityl imidazole was prepared according to literature procedures.[1]

1.2 Instrumentation and Software

^{1}H, ^{13}C, and ^{19}F NMR spectra were recorded on a Varian 400 MHz instrument operating at 399.7770024 MHz. Chemical shifts are reported in ppm relative to $SiMe_4$ and were referenced to residual chloroform-*d* at 7.26 ppm or benzene-d_6 at 7.16 ppm in ^{1}H spectra, and chloroform-*d* at 77.16 ppm or benzene-d_6 at 128.06 ppm in ^{13}C spectra. ^{19}F NMR chemical shifts are referenced to the starting material. Spectra were processed using MestReNova and original files and NMR data can be accessed through Zenodo.[2] ^{1}H NMR data for diamagnetic compounds are reported as follows: chemical shift, multiplicity (s = singlet, d = doublet, t = triplet, q = quartet, p = pentet, br = broad, m = multiplet, app = apparent, obsc = obscured), coupling constants (Hz), integration, assignment. ^{13}C NMR data for diamagnetic compounds are reported as follows: chemical shift, number of protons attached to carbon (e.g. CH_2), assignment.

Elemental analyses were conducted by Robertson Microlit Laboratories, Inc., in Ledgewood, NJ. Infrared spectroscopy was conducted on a Bruker Alpha-II ATR FT-IR spectrometer calibrated with a polystyrene standard, and were processed using the Origin software. The GC-MS separation was performed by a 6890N Agilent gas chromatograph equipped coupled with a 5973 inert MSD operated at 70 eV. A total of 1 μL of sample was injected onto a DB5-MS column with

an i.d. of 0.25 mm, a 10 m guard column and additional functionalized 30 m length for analyte separation.

Single crystals suitable for X-ray diffraction were coated with Paratone-n oil in the glovebox and quickly transferred to a MiTeGen microloop and quickly transferred to the goniometer head of a Bruker D8 Venture Kappa diffractometer with a Mo X-ray tube (λ = 0.71073 Å) and a Cu X-ray tube (λ = 1.54178 Å). The data collection strategy was optimized for completeness and redundancy using the Bruker APEX4 software suite.[3] The space group was identified, and the data were processed using the Bruker SAINT+ program and corrected for absorption using SADABS2016/2.[3,4] The structures were solved using direct methods (SHELXS) completed by subsequent Fourier synthesis and refined by full-matrix least-squares procedures.[5,6] Final refinement was conducted using Olex2.[7] Visualizations were made the Mercury program,[8] or with the online IUCR publishing tools.[9]

All DFT calculations were performed with the ORCA package version 5.0.4 in the gas phase,[10,11] using the San Diego Supercomputing Facility Expanse cluster through NSF ACCESS.[12] Geometry optimizations and single-point calculations were carried out at the B3LYP level of DFT.[13–15] This hybrid functional often outperforms pure gradient-corrected functionals in the accurate representation of transition metal complexes, especially those involving significant metal–ligand covalency.[16] Alrichs' all-electron Gaussian basis sets were employed for all calculations,[17–19] and the triple-ζ basis set def2-TZVP with one set of polarization functions was used to describe metal atoms and all atoms directly coordinated to a metal center. The double-ζ basis set def2-SV(P) was used for all other atoms, which includes one set of polarizing *d*-functionals on all non-hydrogen atoms. Auxiliary basis sets were chosen to match the orbital basis.[20–22] The RIJCOSX approximation was used to accelerate the calculations.[23–25] Representations of canonical orbitals and the corresponding spin density plots were generated with the program *Chimera*.[26]

1.3 Abbreviations

^{Mes}Im = mesityl imidazole, 2,4,6-trimethylphenyl imidazole; [^{Mes}NHC$_2$^{o}Xy][Br]$_2$ = 3,3'-dimesityl-1,1-orthoxylylenediimidazoline-2,2'diylidene dibromide; (^{Mes}NHC$_2$^{o}Xy)Ni(COD) = (η^2-cyclooctadiene)(3,3'-dimesityl-1,1-orthoxylylenediimidazoline-2,2'diylidene)Ni(0); THF = tetrahydrofuran

2. Synthetic Procedures.

2.1 Synthesis of [^{Mes}NHC$_2$^{o}Xy][Br]$_2$ (1)

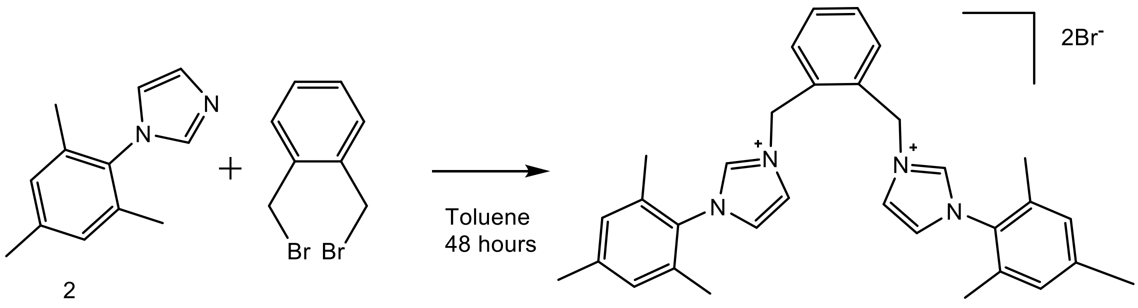

Scheme S1: Synthesis of [^{Mes}NHC$_2$^{o}Xy][Br]$_2$ (1).

Adapted from literature procedure using a different solvent.[27] A 100 mL round bottomed flask was charged with 1.070 g mesityl imidazole (5.369 mmol, 2.5 eq), 0.617 g 1,2-dibromoxylene (2.15 mmol, 1 eq), approximately 10 mL of toluene, and a stir bar. The mixture was then stirred and refluxed for 30 hours at 180 °C. After refluxing, the product was placed in a freezer at 4 °C for 12 hours. The product was then vacuum filtered and washed with cold ethyl acetate. The white powder was then set aside to dry for 2 days. After complete drying the sample was weighed giving a product of 1.49 g (99.8%) of white powder identified as [^{Mes}NHC$_2$^{o}Xy][Br]$_2$.

^{1}H NMR (399.777 MHz, $CDCl_3$, 25 °C): δ = 2.09 (s, 12H; Mes *o*-CH_3), 2.32 (s, 6H; Mes *p*-CH_3), 6.39 (s, 4H; ^{o}Xy-CH_2), 6.98 (s, 4H; Mes-CH), 7.18 (s, 2H; CH-Im), 7.37 (m, 4H, ^{o}Xy), 8.21 (s, 2H; CH-Im), 10.26 (s, 2H; CH-Im).

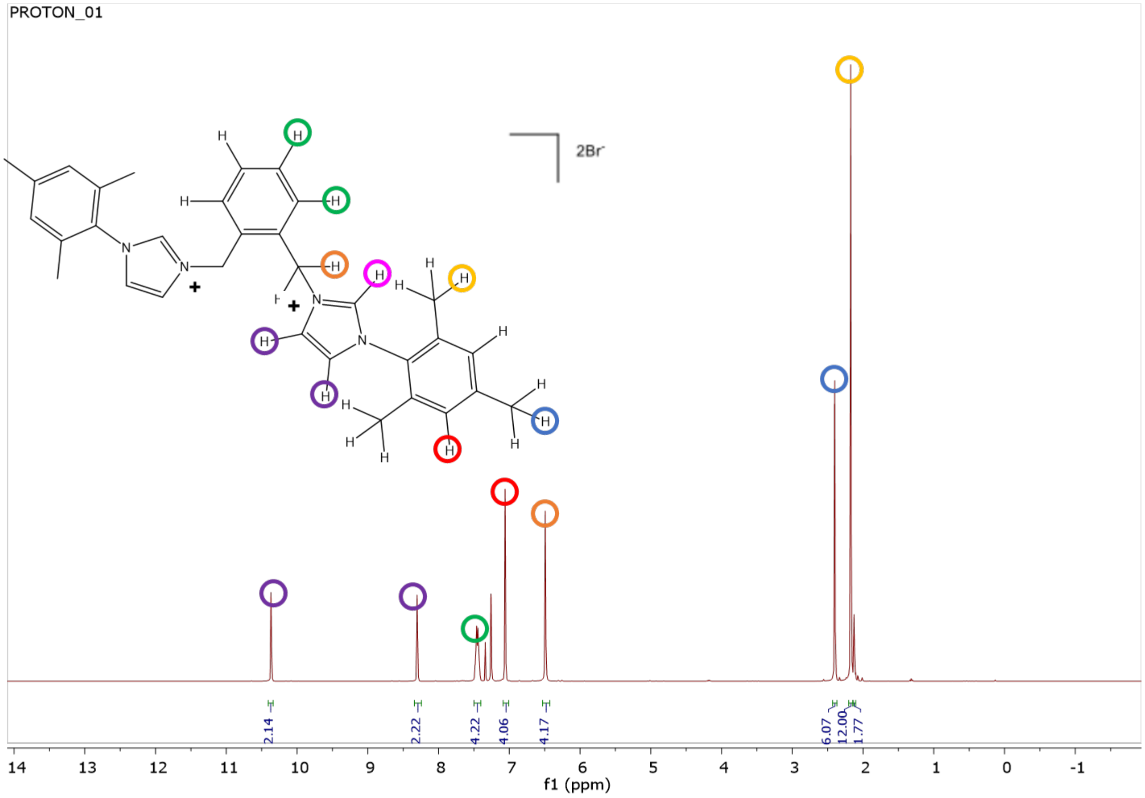

Figure S1. ^{1}H NMR of [^{Mes}NHC$_2$^{o}Xy][Br]$_2$ **1** in $CDCl_3$ at 25 ^oC.

2.2 Synthesis of (^{Mes}NHC$_2$^{o}Xy)Ni(η^2-COD) (2):

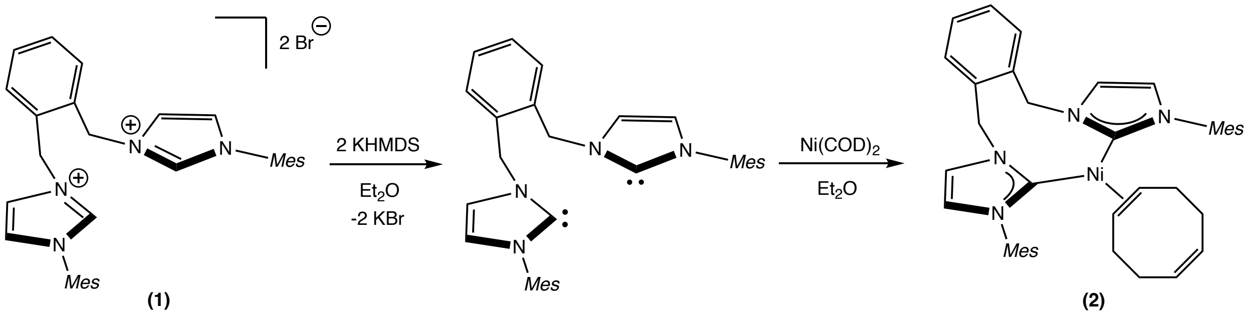

Scheme S2: Synthesis of (^{Mes}NHC$_2$^{o}Xy)Ni(η^2-COD) (**2**).

A 20 mL glass scintillation vial was charged with 0.4003 g (6.29×10^{-4} mol, 1 eq) of [^{Mes}NHC$_2$^{o}Xy][Br]$_2$ approximately 7 mL of Et_2O or THF and a stir bar. While stirring, 2.2 mL (1.54×10^{-2} mol, 2.4 eq) of 0.7 M potassium bis(trimethylsilyl)amide (KHMDS) was added drop

wise to the solution. After 5 hours, the solution was filtered over a pad of celite and the raspberry filtrate was collected. The filtrate was transferred to a vial with a stir bar. While stirring, 0.1530 g ($5.56x10^{-4}$ mol, 0.9 eq) of $Ni(COD)_2$ was added. The next day, the solvent was removed in vacuo and approximately 10 mL of hexanes was added, followed by vigorous stirring. The orange precipitate (197.9 mg, 55.4% yield) was collected on a small frit via vacuum filtration and identified as ($^{Mes}NHC_2{}^{o}Xy$)Ni(η^2-COD). The filtrate was stored in freezer for a second crop. A single crystal suitable for X-ray diffraction was grown from a saturated solution in benzene at 4°C, following the reaction in THF.

^{1}H NMR (399.777 MHz, THF-d_8: δ = 3.58, 25 °C): δ = 1.92 (s, 12H; Mes *o*-C*H*$_3$), 2.31 (s, 6H; Mes *p*- C*H*$_3$), 4.8 (1H, m; COD-C*H*$_2$), 5.09 (1H, d, 13.8 Hz; ^{o}Xy-C*H*$_2$), 5.17 (1H, d, 14.3 Hz; ^{o}Xy-C*H*$_2$), 5.36 (1H, m; COD-C*H*), 5.59 (1H, m; COD-C*H*), 5.68 (1H, m; ^{o}Xy-C*H*$_2$), 6.28 (1H, dd, 14.3 Hz, 2.3 Hz; ^{o}Xy-C*H*$_2$), 6.53 (1H, m; ^{o}Xy-C*H*), 6.56 (1H dd, 5 Hz; ^{o}Xy-C*H*), 6.68 (1H, m; ^{o}Xy-C*H*), 6.79 (2H, d, 0.8 Hz; NHC-C*H*), 6.87 (2H, d, 0.8 Hz; NHC-C*H*), 6.89 (s, 4H; Mes-C*H*), 7.37 (1H, d, 11.7 Hz; ^{o}Xy-C*H*), 7.44 (1H, m; Ni-COD-C*H*). Uneven background and significant overlapping signals interfered with fully accurate integration and assignments.

^{13}C NMR (101 MHz, THF-d_8: δ = 3.58, 25 °C): δ = 17.79 (Mes *o*-CH$_3$), 20.91 (Mes *p*-*C*H$_3$), 29.0 (COD-*C*H$_2$), 32.7 (COD-*C*H$_2$), 52.58 (^{o}Xy-*C*H$_2$), 53.92 (^{o}Xy-*C*H$_2$), 122.51 (NHC-*C*H), 126.99 (NHC-*C*H), 128.9 (Mes-*C*H), 129.04 (^{o}Xy), 129.28 (^{o}Xy), 130.0 (NHC-*C*H), 131.6 (COD-*C*H), 132.25 (COD-*C*H), 136.4 (Mes-*C*), 136.8 (NHC-*C*), 138.4 (Mes-*C*), 139.2 (NHC-*C*).

Elemental Analysis for $C_{40}H_{46}Ni_1N_4$, MW = 641.53: Calculated: C 74.89% H 7.23% N 8.73%. Could not be obtained because sample reacts violently with air.

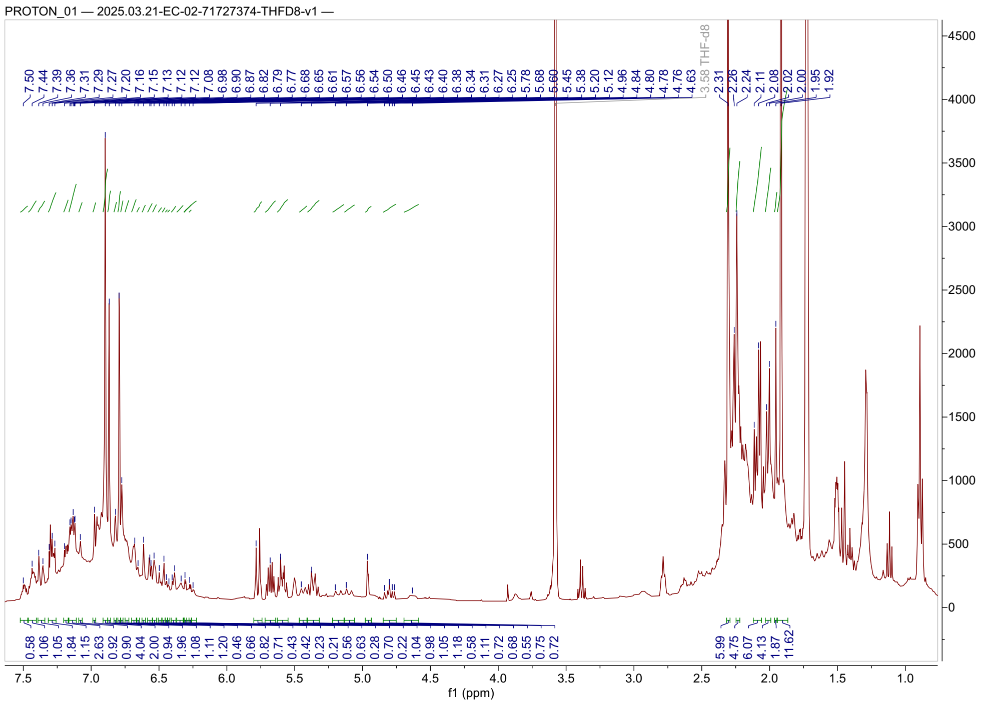

Figure S2. ^{1}H NMR of (^{Mes}NHC$_2$^{o}Xy)Ni(η^2-COD) **2** in THF-d_8 at 23 ^{o}C.

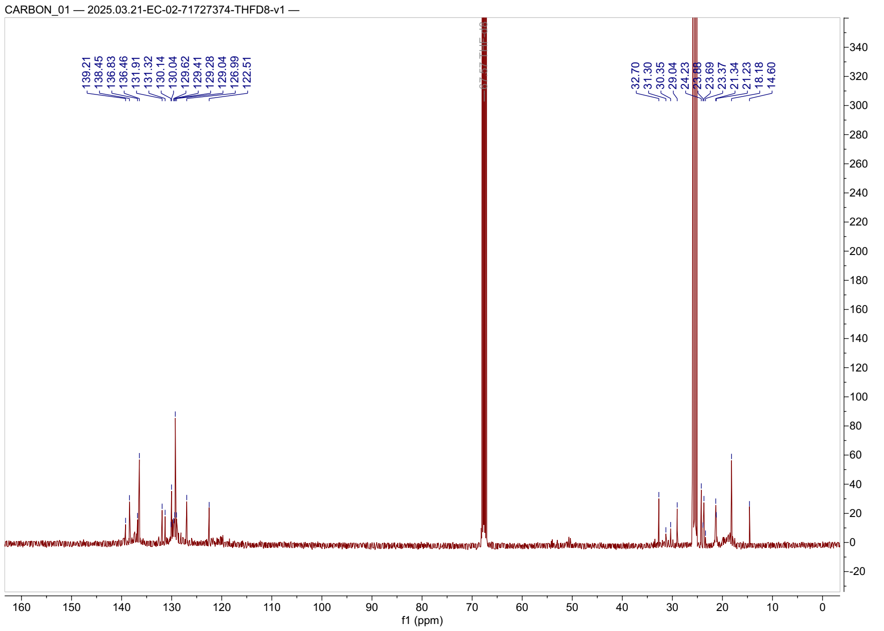

Figure S3. ^{13}C NMR of (^{Mes}NHC$_2$^{o}Xy)Ni(η^2-COD) **2** in THF-d_8 at 23 ^{o}C.

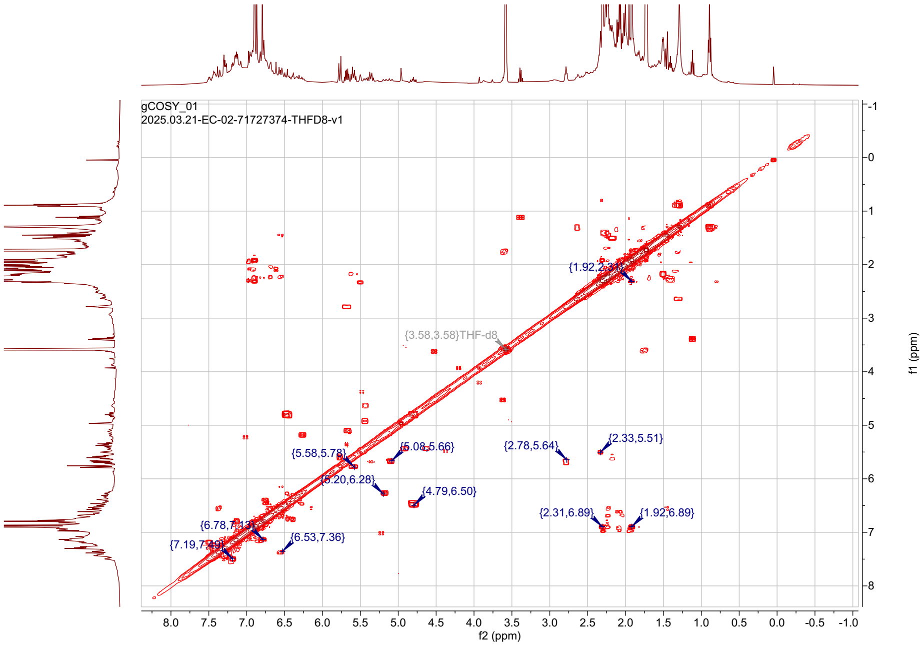

Figure S4. ^{1}H-^{1}H COSY NMR of ($^{Mes}NHC_2{}^{o}Xy$)Ni(η^2-COD) **2** in THF-d_8 at 23 °C.

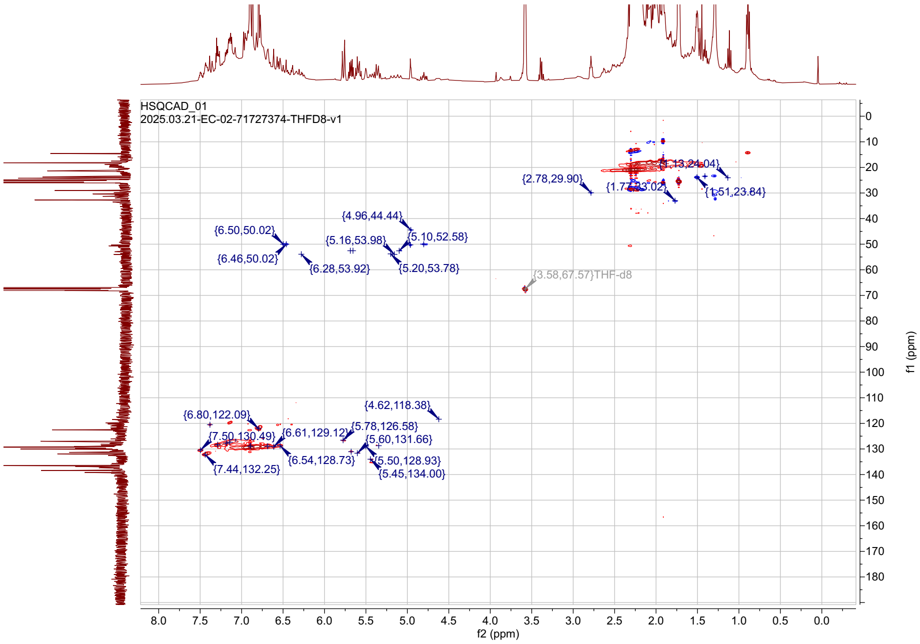

Figure S5. ^{1}H-^{13}C HSQC NMR of ($^{Mes}NHC_2{}^{o}Xy$)Ni(η^2-COD) **2** in THF-d_8 at 23 °C.

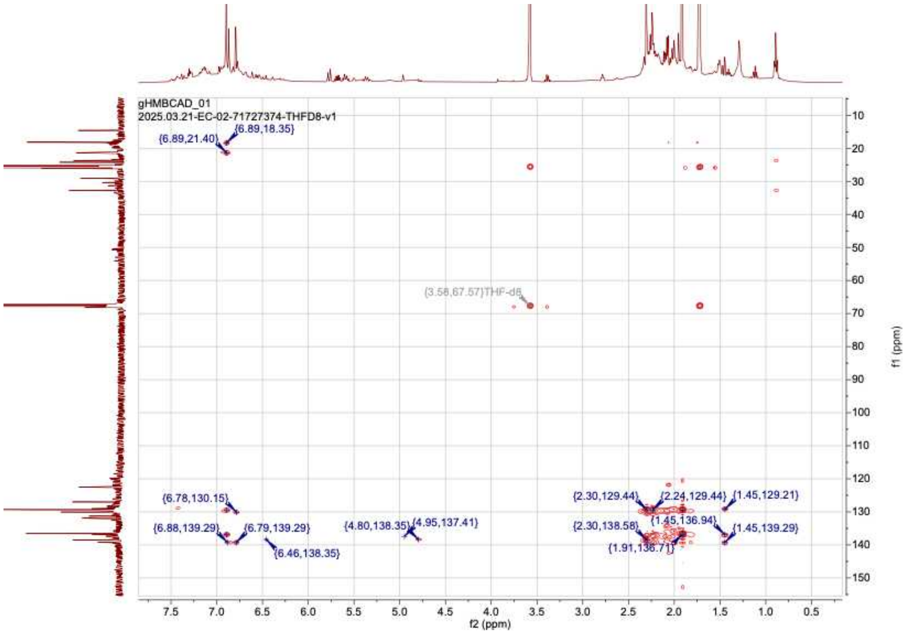

Figure S6. ^{1}H-^{13}C HMBC NMR of ($^{Mes}NHC_2{}^{o}Xy$)Ni(SO_2) **3** in THF-d_8 at 23 °C. In particular, this enabled identification of the signals at 139 ppm and 136 ppm as the NHC carbene carbons due to correlations with the other NHC carbons. Efforts to measure NMR of isolated crystals were not successful, so some starting material *p*-toluene sulfonyl fluoride and hexane are present.

2.3 Synthesis of ($^{Mes}NHC_2{}^{o}Xy$)Ni(SO_2) (3)

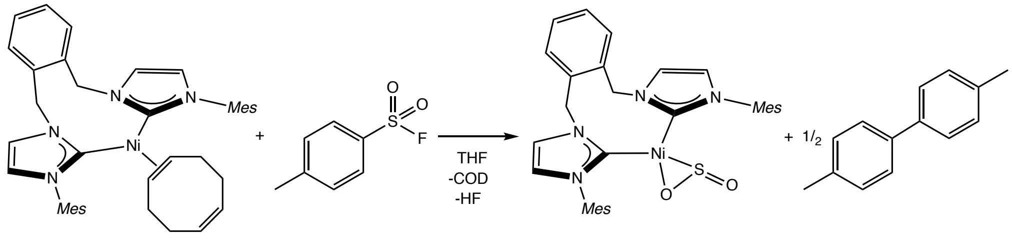

Scheme S3: Synthesis of ($^{Mes}NHC_2{}^{o}Xy$)Ni(SO_2) (**3**).

HAZARD STATEMENT: A byproduct of this reaction is HF, which presents a significant chemical hazard, so appropriate safety measures are imperative. A 20 mL glass scintillation vial was charged with 0.106 g (0.000165 mol, 1 eq.) of ($^{Mes}NHC_2{}^{o}Xy$)Ni(η^2-COD), approximately 5 mL of diethyl ether, and a stir bar. The solution was stirred and 0.0310 g (0.000178 mol, 1.07 eq.) of *p*-toluene sulfonyl fluoride was added. A quick color change to brown was observed and the solution was allowed to stir for 2 days. The precipitate was collected via vacuum filtration and

dried, yielding 0.0677 g (0.000113 mol, 68%) of a brown solid identified as (^{Mes}NHC$_2$^{o}Xy)Ni(SO$_2$). A single crystal suitable for X-ray diffraction was grown from a saturated solution in benzene at 4°C. These crystals were also suitable for FTIR analysis. Efforts to measure NMR of isolated crystals were not successful, so some starting material *p*-toluene sulfonyl fluoride and hexane are present.

^{1}H NMR (399.777 MHz, THF-d_8: δ = 3.58, 25 °C): δ = 1.92 (s, 12H, Mes *o*-C*H*$_3$), 2.30 (s, 6H, Mes *p*-C*H*$_3$), 5.35 (1H, q, 8-9Hz, ^{o}Xy-C*H*$_2$), 5.61 (1H, quintet, 7-9Hz, ^{o}Xy-C*H*$_2$), 5.69 (1H, quintet, 5Hz, ^{o}Xy-C*H*$_2$), 5.77 (1H, d, 10Hz, ^{o}Xy-C*H*$_2$), 6.02, 6.25, 6.61 (overlapping peaks, NHC-C*H*)*, 6.79 (2-3H, s, ^{o}Xy-C*H*), 6.87 (2-3H, s, overlapping, ^{o}Xy-C*H*), 6.90 (s, Mes-C*H*).

*Uneven background and significant overlapping signals interfered with fully accurate integration and assignments.

^{13}C NMR (101 MHz, THF-d_8: δ = 67.57, 25 °C): δ = 139.20 (NHC-*C*), 138.47 (Mes-*C*), 136.80 (NHC-*C*), 136.43 (Mes-*C*), 131.92 (oXy-*C*), 131.3 (^{o}Xy-*C*H$_2$), 130.04 (^{o}Xy-*C*), 129.63 (Mes-*C*H), 129.0 (^{o}Xy-*C*H$_2$), 122.53 (^{o}Xy-*C*H), 21.12 (Mes-*p*-*C*H$_3$), 18.20 (Mes-*o*-*C*H$_3$).

^{19}F NMR (376 MHz, THF-d_8, 25 °C): δ = 67.22 (s, SO$_2$ starting material, reference).

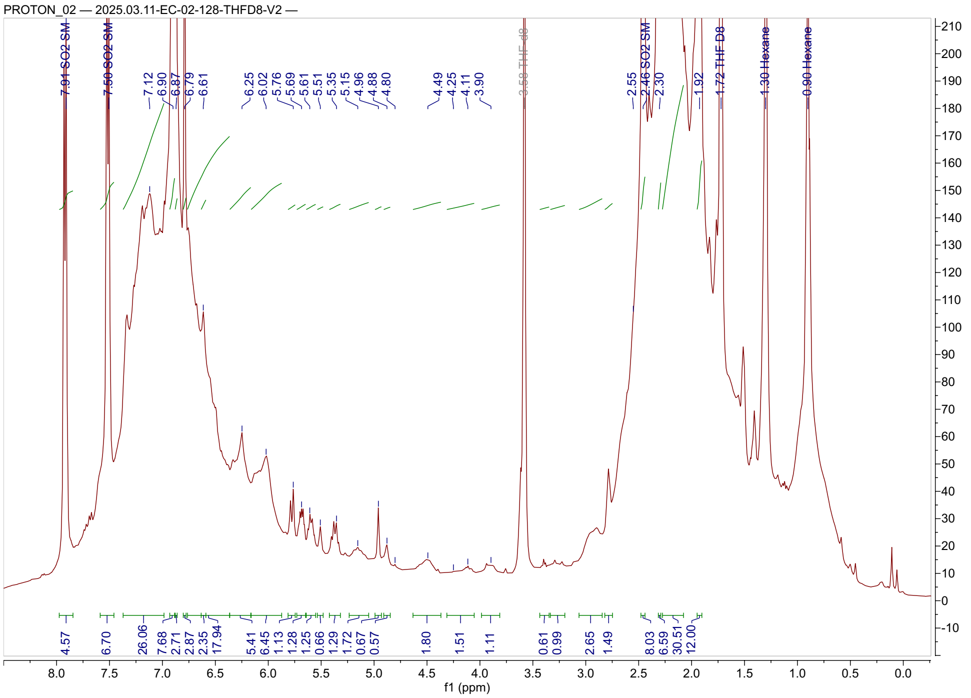

Figure S7. ^{1}H NMR of (^{Mes}NHC$_2$^{o}Xy)Ni(SO_2) **3** in THF-d_8 at 25 °C. Efforts to measure NMR of isolated crystals were not successful, so some starting material *p*-toluene sulfonyl fluoride and hexane are present.

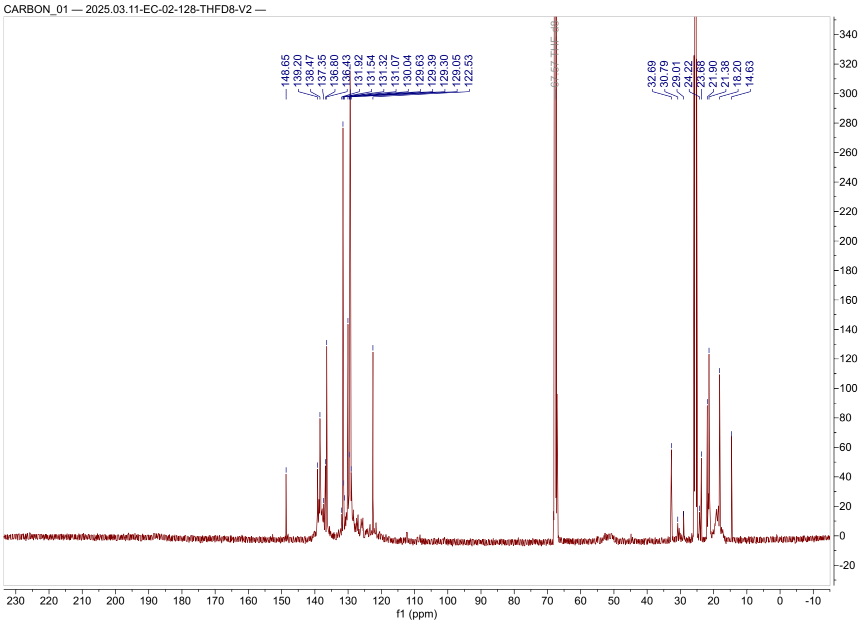

Figure S8. ^{13}C NMR of (^{Mes}NHC$_2$^{o}Xy)Ni(SO_2) **3** in THF-d_8 at 25 °C. Efforts to measure NMR of isolated crystals were not successful, so some starting material *p*-toluene sulfonyl fluoride and hexane are present.

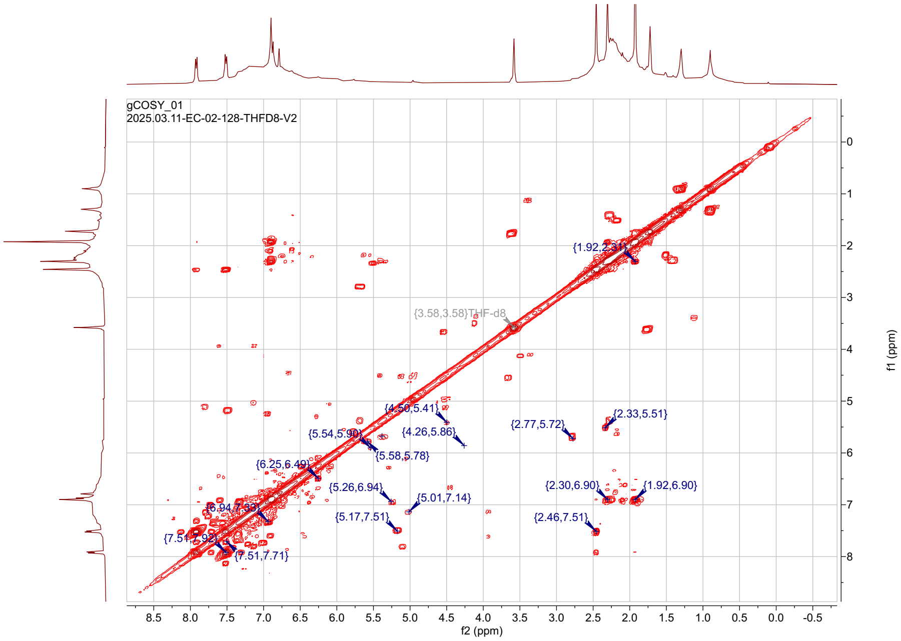

Figure S9. ^{1}H-^{1}H COSY NMR of (^{Mes}NHC$_2$^{o}Xy)Ni(SO$_2$) **3** in THF-*d*$_8$ at 23 °C. Efforts to measure NMR of isolated crystals were not successful, so some starting material *p*-toluene sulfonyl fluoride and hexane are present.

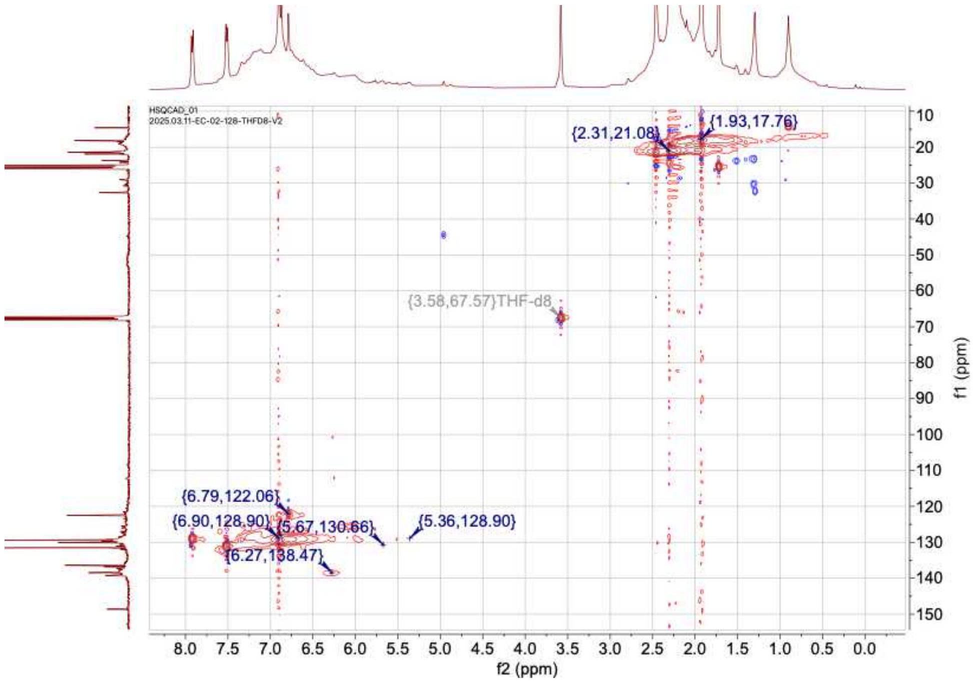

Figure S10. ^{1}H-^{13}C HSQC NMR of (^{Mes}NHC$_2$^{o}Xy)Ni(SO$_2$) **3** in THF-*d*$_8$ at 23 °C. Efforts to measure NMR of isolated crystals were not successful, so some starting material *p*-toluene sulfonyl fluoride and hexane are present.

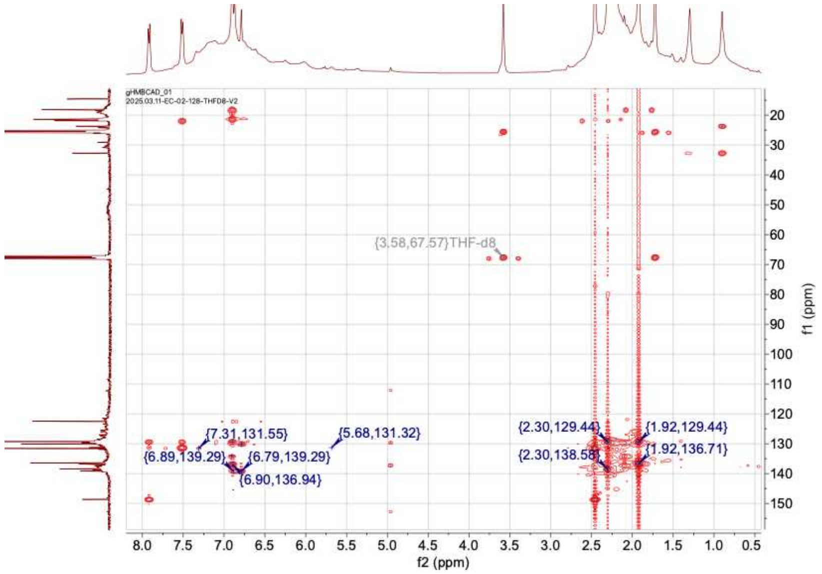

Figure S11. ^{1}H-^{13}C HMBC NMR of (^{Mes}NHC$_2$^{o}Xy)Ni(SO_2) **3** in THF-d_8 at 23 °C. In particular, this enabled identification of the signals at 139 ppm and 136 ppm as the NHC carbene carbons due to correlations with the other NHC carbons. Efforts to measure NMR of isolated crystals were not successful, so some starting material *p*-toluene sulfonyl fluoride and hexane are present.

3. Additional characterization, including NMR, IR, GC-MS

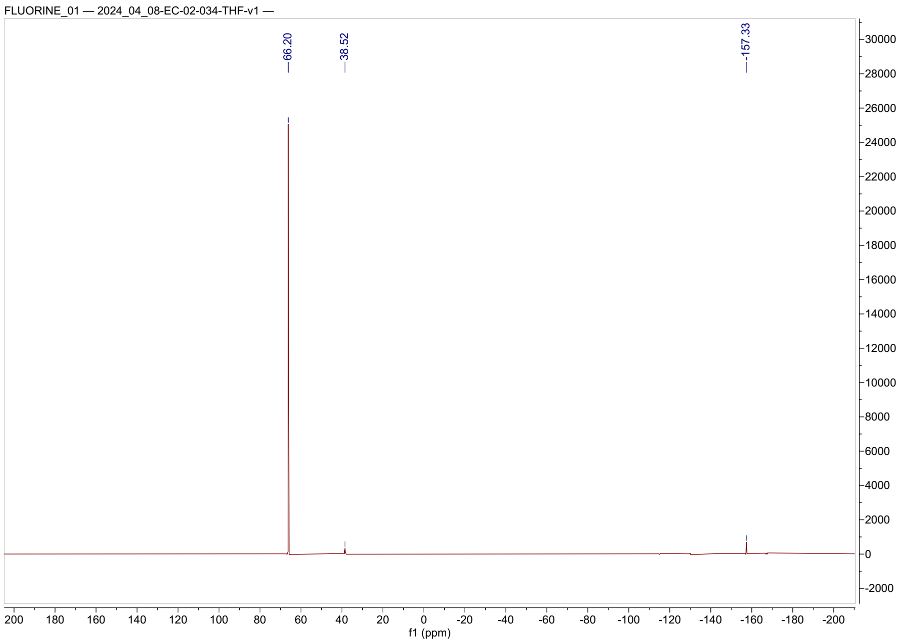

Figure S12. ^{19}F NMR of crude reaction mixture of (^{Mes}NHC$_2$^{o}Xy)Ni(SO_2) **3** in THF-d_8 at 25 °C.

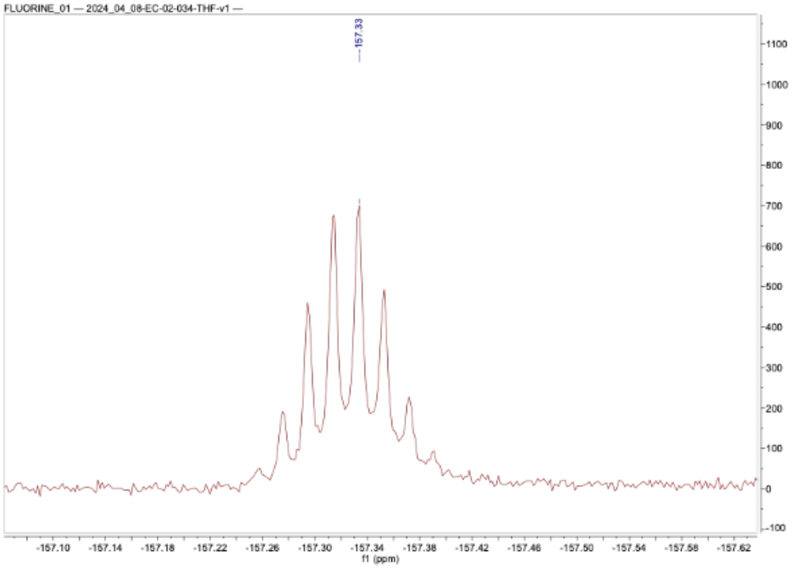

Figure S13. ^{19}F NMR of crude reaction of ($^{Mes}NHC_2{}^oXy$)Ni(SO_2) **3** in THF-d_8 at 25 °C indicating the presence of a fluorosilane. This is likely a result of residual HMDS reacting with F^- generated by the reaction.

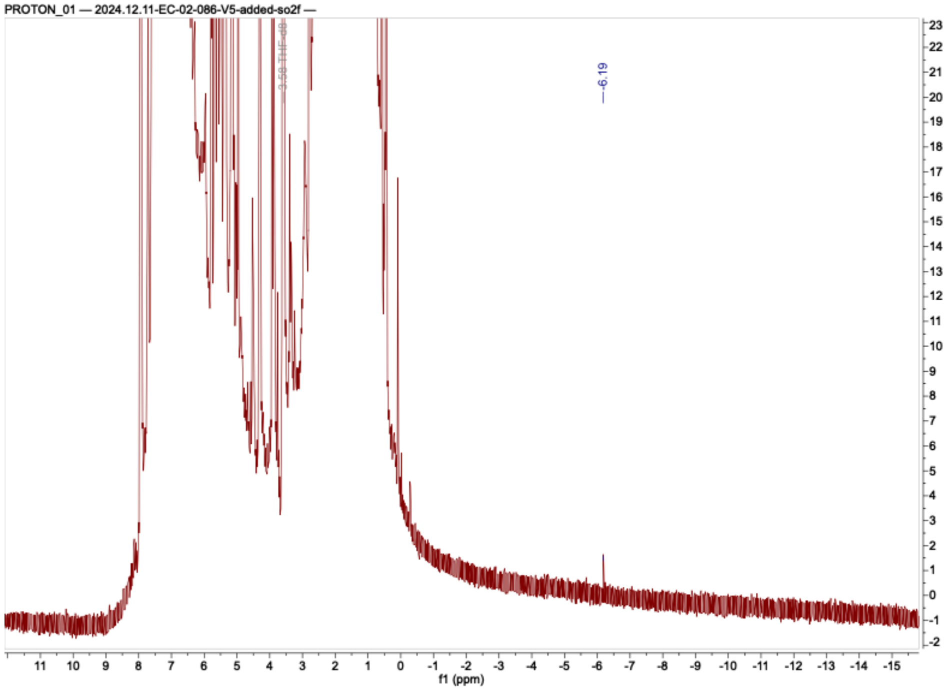

Figure S14. ^1H NMR of reaction mixture in J-Young Tube from ($^{Mes}NHC_2{}^oXy$)Ni(COD) **2** and *p*-toluene sulfonyl fluoride in THF-d_8 at 25 °C indicating the presence of HF at -6.19 ppm.

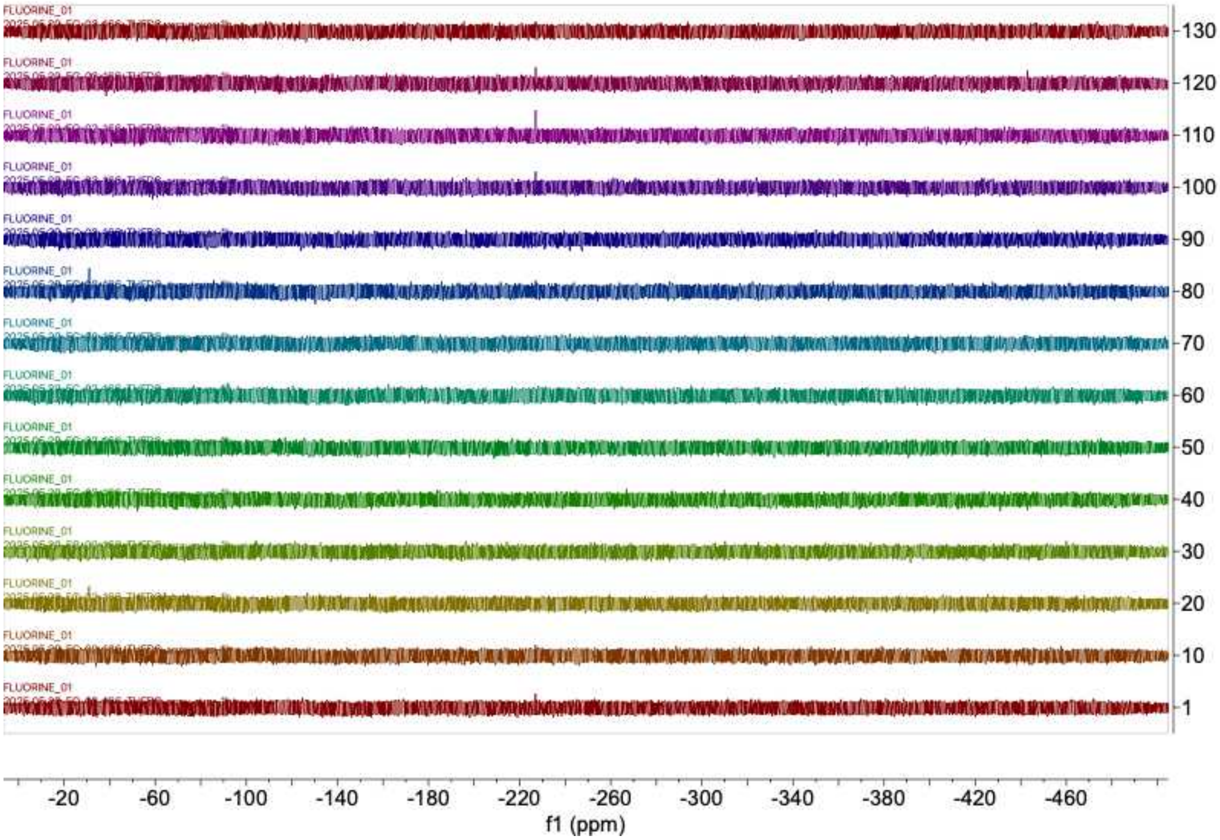

Figure S15. An array of ^{19}F NMR spectra over the course of 1 hour of a reaction mixture in J-Young Tube from ($^{Mes}NHC_2{}^{o}Xy$)Ni(COD) **2**, *p*-toluene sulfonyl fluoride, and CsCl in an attempt to trap a nickel fluoride in THF-d_8 at 25 °C. The spectra indicate the likely formation of SO_2F_2 at -31 ppm and F- formation at -227 ppm.

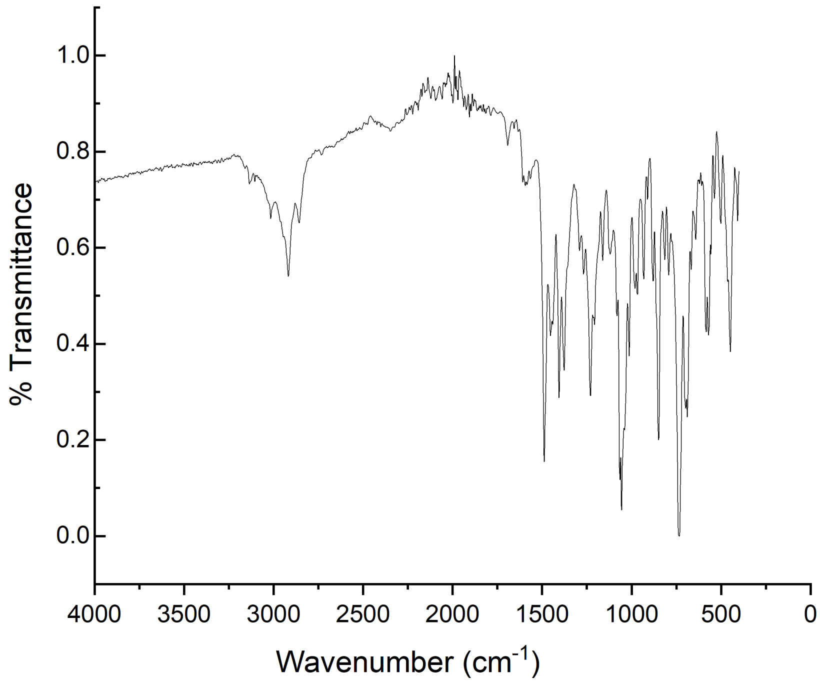

Figure S16. ATR FTIR IR spectrum of (^{Mes}NHC$_2$^{o}Xy)Ni(SO$_2$) **3.**

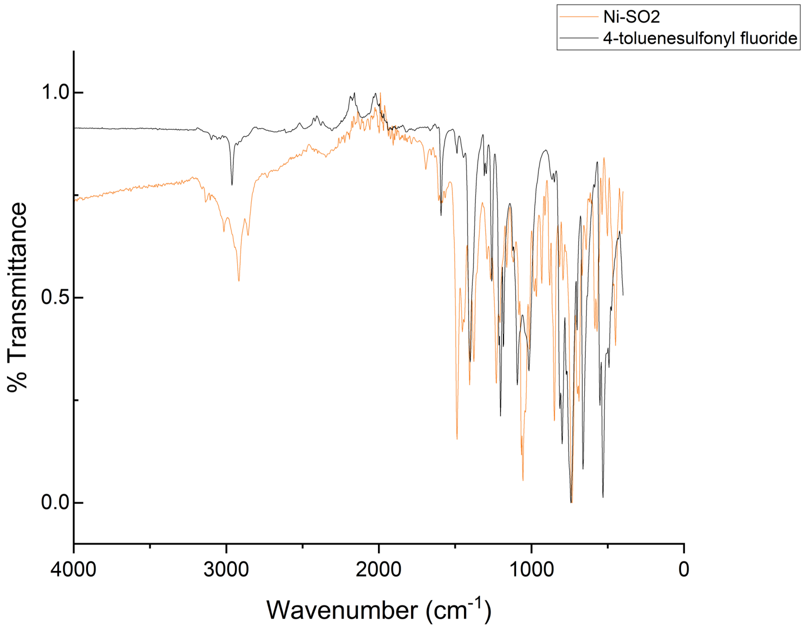

Figure S17. ATR FTIR spectra of (^{Mes}NHC$_2$^{o}Xy)Ni(SO$_2$) **3** (orange) and 4-toluenesulfonyl fluoride (black).

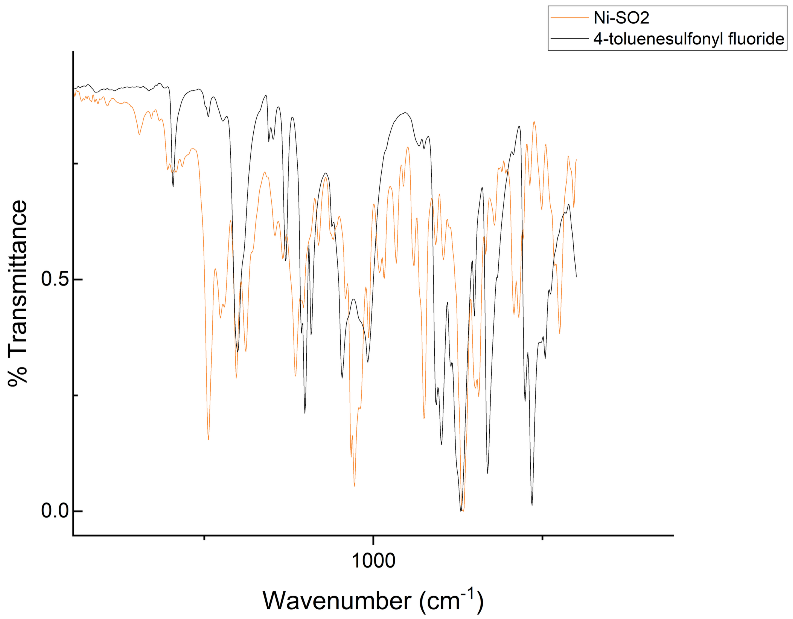

Figure S18. IR spectra of (^{Mes}NHC$_2$^{o}Xy)Ni(SO$_2$) **3** (orange) and 4-toluenesulfonyl fluoride (black, 1700 – 700 cm^{-1}).

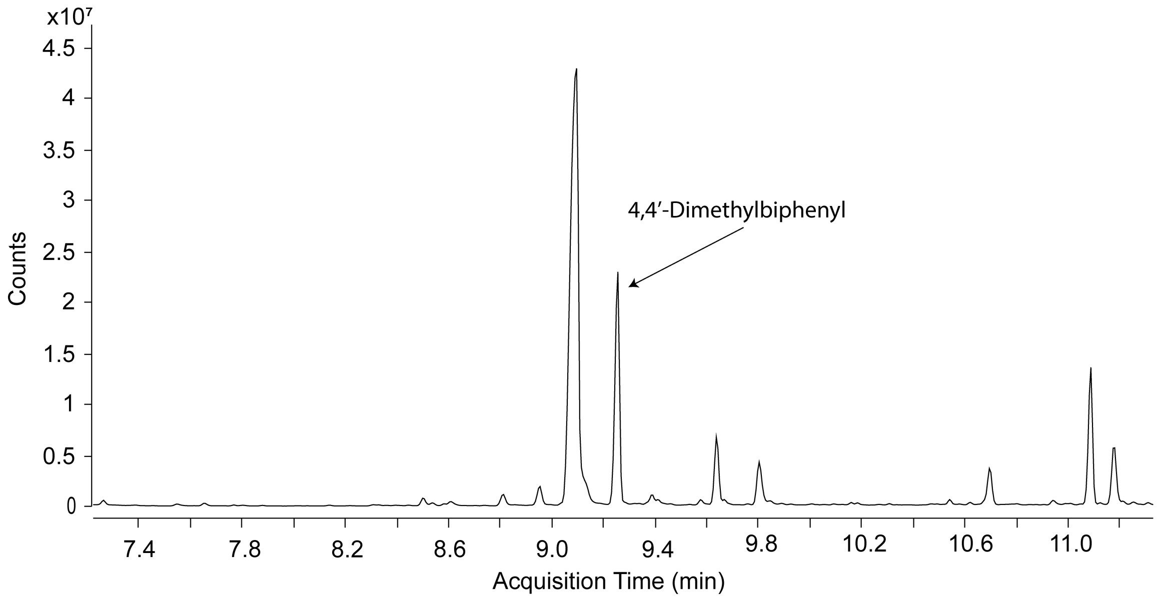

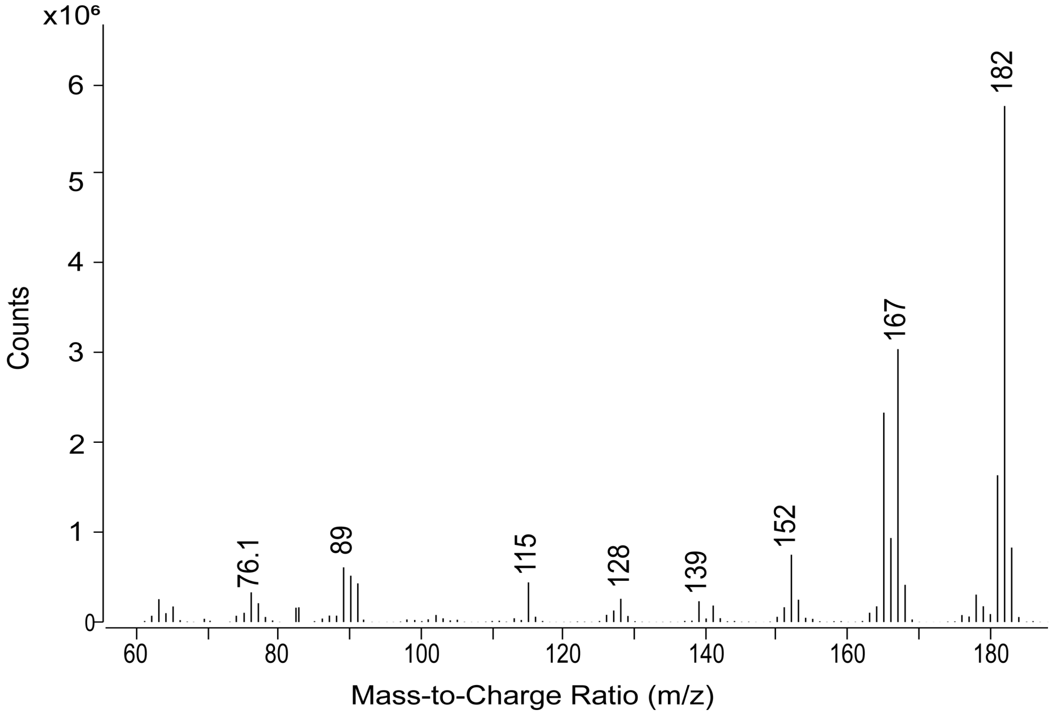

Figure S19. Partial chromatogram of reaction mixture for forming ($^{Mes}NHC_2{}^oXy$)Ni(SO_2) **3** in THF (top) resulting from an NMR tube reaction, and the extracted mass spectrum of the peak at 9.25 min was queried with the NIST Library by AMDIS and putatively identified as 4,4'-dimethybiphenyl (bottom).

4. Crystallographic Data

4.1 [^{Mes}NHC$_2$^{o}Xy][Br]$_2$

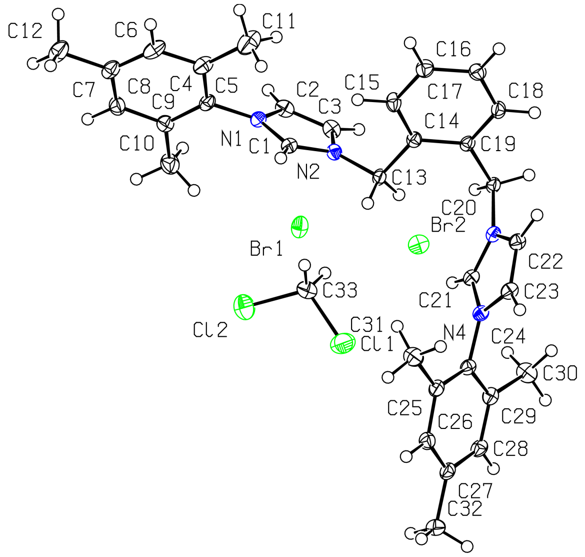

Figure S20. Representation of the solid state structure of [^{Mes}NHC$_2$^{o}Xy][Br]$_2$ at 50% ellipsoids.

Table S1. Crystal data for [^{Mes}NHC$_2$^{o}Xy][Br]$_2$.

	jb01067_0m
Crystal data	
Chemical formula	2(Br)·CH_2Cl_2·$C_{32}H_{36}N_4$
M_r	721.39
Crystal system, space group	Triclinic, $P\bar{1}$
Temperature (K)	107
a, *b*, *c* (Å)	8.9232 (2), 11.5727 (4), 17.3718 (5)
a, b, g (°)	89.837 (1), 84.070 (1), 68.491 (1)
V (Å^3)	1658.86 (8)
Z	2
Radiation type	Mo *K*a
m (mm^{-1})	2.63
Crystal size (mm)	0.3 × 0.13 × 0.05
Data collection	

Diffractometer	Bruker Venture Kappa D8
Absorption correction	Multi-scan *SADABS2016*/2 (Bruker,2016/2) was used for absorption correction. wR2(int) was 0.0871 before and 0.0559 after correction. The Ratio of minimum to maximum transmission is 0.8865. The l/2 correction factor is Not present.
T_{min}, T_{max}	0.661, 0.746
No. of measured, independent and observed [*I* > 2s(*I*)] reflections	60114, 10144, 7531
R_{int}	0.066
(sin q/l)$_{max}$ (Å^{-1})	0.715
Refinement	
R[F^2 > 2s(F^2)], *wR*(F^2), *S*	0.040, 0.085, 1.02
No. of reflections	10144
No. of parameters	392
H-atom treatment	H atoms treated by a mixture of independent and constrained refinement
D⟩$_{max}$, D⟩$_{min}$ (e Å^{-3})	0.81, -0.48

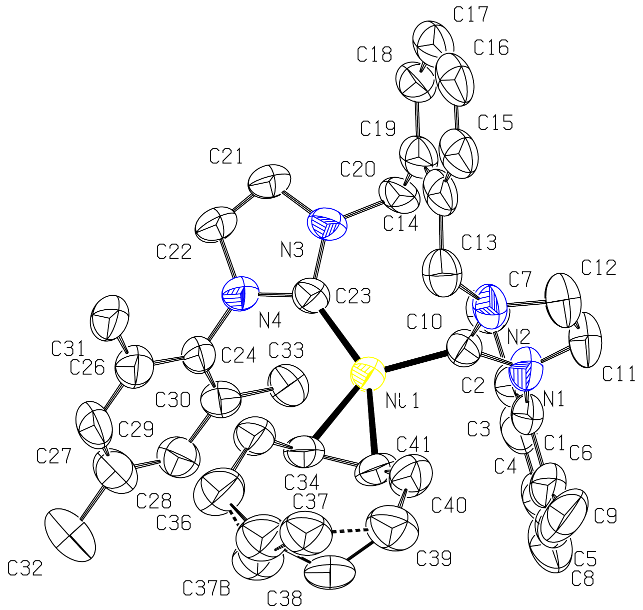

Figure S21. Representation of the solid state structure of (^{Mes}NHC$_2$^{o}Xy)Ni(η^2-COD) at 50% ellipsoids with hydrogen atoms omitted for clarity.

Table S2. Crystal data for ($^{Mes}NHC_2^{o}Xy$)Ni(η^2-COD).

	cu_ec01012b_autostructure_0m
Crystal data	
Chemical formula	C40H48N4Ni·0.5[C6H6]
Mr	682.58
Crystal system, space group	Monoclinic, C2/c
Temperature (K)	104
a, b, c (Å)	26.1460 (4), 18.7412 (3), 16.8020 (3)
β (°)	114.553 (1)
V (Å3)	7488.6 (2)
Z	8
Radiation type	Cu Kα
μ (mm−1)	1.00
Crystal size (mm)	0.1 × 0.1 × 0.1
Data collection	
Diffractometer	Bruker APEX-II CCD
Absorption correction	Multi-scan<br>SADABS2016/2 (Bruker,2016/2) was used for absorption correction. wR2(int) was 0.1393 before and 0.0818 after correction. The Ratio of minimum to maximum transmission is 0.8421. The λ/2 correction factor is Not present.
Tmin, Tmax	0.623, 0.752
No. of measured, independent and observed [I > 2σ(I)] reflections	51790, 5972, 3444
Rint	0.111
(sin θ/λ)max (Å−1)	0.575
Refinement	
R[F2 > 2σ(F2)], wR(F2), S	0.070, 0.233, 1.04
No. of reflections	5972
No. of parameters	431
No. of restraints	116
H-atom treatment	H-atom parameters constrained
	w = 1/[σ2(Fo2) + (0.108P)2 + 20.9609P]<br>where P = (Fo2 + 2Fc2)/3

Δρmax, Δρmin (e Å−3)	0.41, −0.54

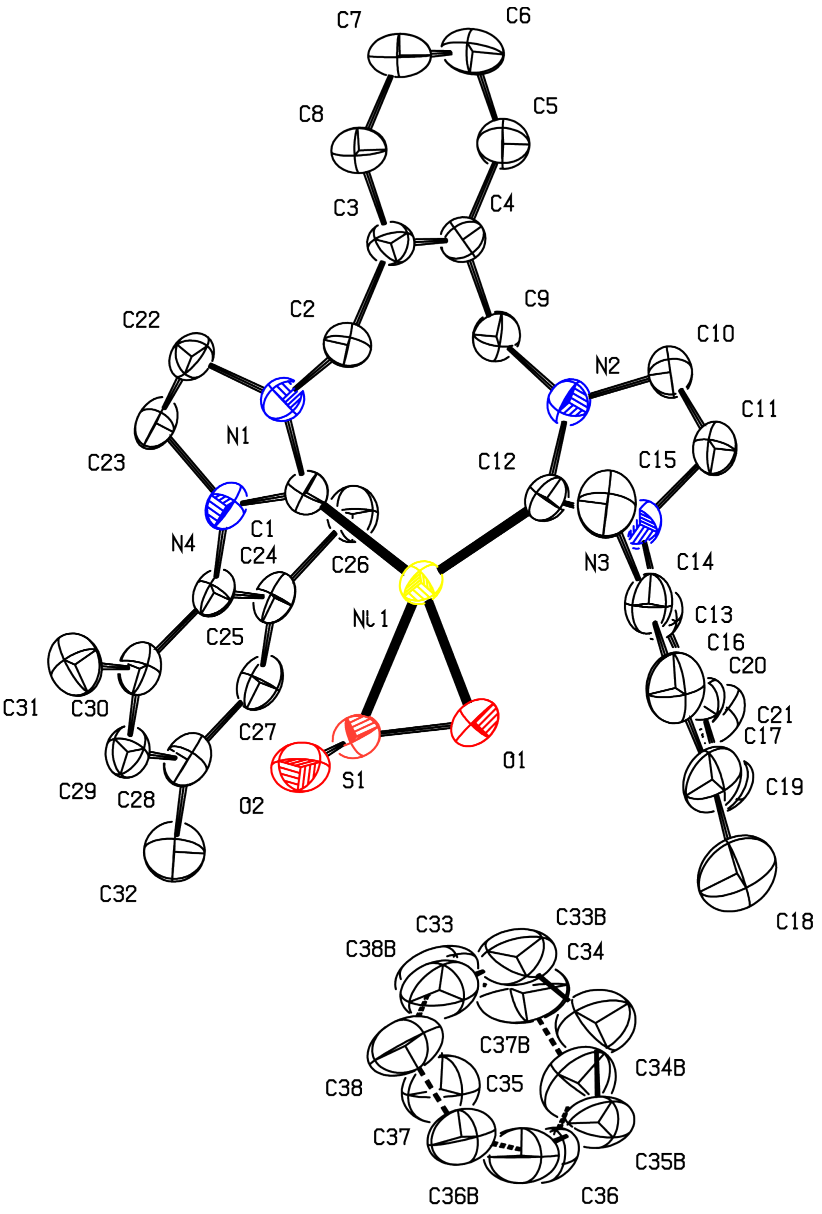

Figure S22. Representation of the solid state structure of (^{Mes}NHC$_2$^{o}Xy)Ni(SO$_2$) and co-crystallized benzene at 50% ellipsoids with hydrogen atoms omitted for clarity.

Table S3. Crystal data for (^{Mes}NHC$_2$^{o}Xy)Ni(SO$_2$).

	mo_ec01116b_2_0m
Crystal data	
Chemical formula	$C_{32}H_{33}N_4NiO_2S{\cdot}C_6H_6{\cdot}1[C6H_6]$
M_r	752.61
Crystal system, space group	Monoclinic, *C*2/*c*
Temperature (K)	200
a, *b*, *c* (Å)	22.8902 (6), 12.2374 (4), 26.6919 (8)
b (°)	94.875 (1)
V (Å^3)	7449.8 (4)
Z	8
Radiation type	Mo *K*a
m (mm^{-1})	0.62
Crystal size (mm)	0.3 × 0.15 × 0.15
Data collection	
Diffractometer	Bruker *APEX*-II CCD
Absorption correction	Multi-scan *SADABS2016*/2 (Bruker,2016/2) was used for absorption correction. wR2(int) was 0.0920 before and 0.0574 after correction. The Ratio of minimum to maximum transmission is 0.8992. The l/2 correction factor is Not present.
T_{min}, T_{max}	0.671, 0.746
No. of measured, independent and observed [*I* > 2s(*I*)] reflections	105325, 10041, 8348
R_{int}	0.050
(sin q/l)$_{max}$ (Å^{-1})	0.686
Refinement	
$R[F^2 > 2s(F^2)]$, $wR(F^2)$, S	0.040, 0.120, 1.06
No. of reflections	10041
No. of parameters	452
No. of restraints	210
H-atom treatment	H-atom parameters constrained
D〉$_{max}$, D〉$_{min}$ (e Å^{-3})	0.30, -0.34

5. Computational Analyses

All input, output, and xyz files can be found at Zenodo.org.[2]

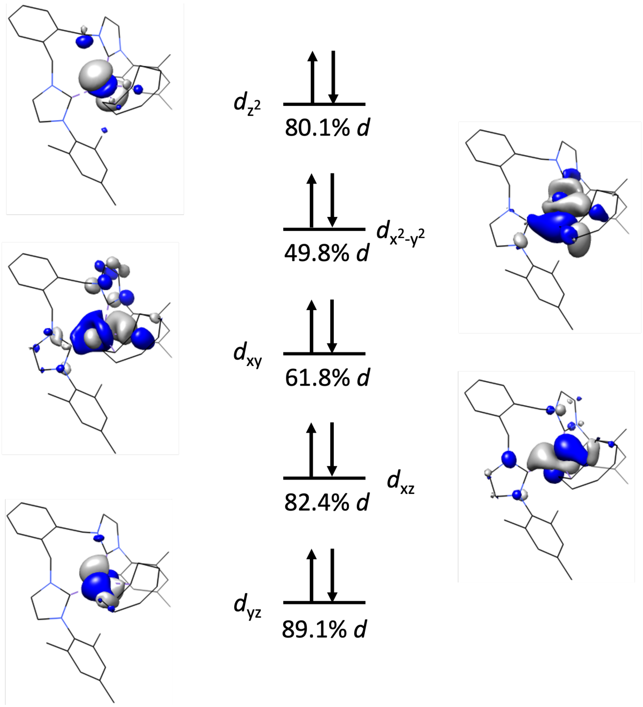

Figure S23. Qualitative *d*-orbital splitting diagram for $(^{Mes}NHC_2{}^{o}Xy)Ni(\eta^2\text{-COD})$ resulting from a B3LYP geometry optimization.

Table S4. Selected experimental versus calculated bond distances (Å) and angles (°) for $(^{Mes}NHC_2{}^{o}Xy)Ni(SO_2)$ resulting from geometry optimization.

	**Experimental**	**Calculated**
Ni1-S1	2.1413(5)	2.172
Ni1-O1	1.9263(14)	1.926
Ni1-C1	1.8942(16)	1.896
Ni1-C12	1.9541(18)	1.976
S1-O1	1.5428(15)	1.559
S1-O2	1.4848(16)	1.492
Ni1-S1-O1	60.49(5)	59.5
Ni1-S1-O2	113.12(7)	109.8
O1-S1-O2	112.71(10)	114.2
C1-Ni1-C12	108.57(7)	110.0
S1-Ni1-C12	146.68(5)	143.1

6. References

(1) Liu, J.; Chen, J.; Zhao, J.; Zhao, Y.; Li, L.; Zhang, H. A Modified Procedure for the Synthesis of 1-Arylimidazoles. *Synthesis* **2003**, *2003* (17), 2661–2666. https://doi.org/10.1055/s-2003-42444.

(2) Stieber, S. C. E.; Chavarin, E. B.; Marr, Z. Y.; Brannon, J. P.; Antonini Bertolazzo, A.; Barding, G. A. Experimental and Computational Data for Sulfonyl Fluoride Activation via S-F and C-S Bond Cleavage by a Ni(0) Bidentate N-Heterocyclic Carbene Complex, 2025. https://doi.org/10.5281/zenodo.15083911.

(3) Bruker. APEX4 and SAINT, 2016.

(4) Krause, L.; Herbst-Irmer, R.; Sheldrick, G. M.; Stalke, D. *J. Appl. Cryst.* **2015**, *48*, 3–10.

(5) Sheldrick, G. M. Crystal Structure Refinement with SHELXL. *Acta Cryst C* **2015**, *71* (1), 3–8. https://doi.org/10.1107/S2053229614024218.

(6) Sheldrick, G. M. SHELXT – Integrated Space-Group and Crystal-Structure Determination. *Acta Cryst A* **2015**, *71* (1), 3–8. https://doi.org/10.1107/S2053273314026370.

(7) Dolomanov, O. V.; Blake, A. J.; Champness, N. R.; Schröder, M. OLEX: New Software for Visualization and Analysis of Extended Crystal Structures. *J Appl Cryst* **2003**, *36* (5), 1283–1284. https://doi.org/10.1107/S0021889803015267.

(8) Macrae, C. F.; Edgington, P. R.; McCabe, P.; Pidcock, E.; Shields, G. P.; Taylor, R.; Towler, M.; Streek, J. van de. Mercury: Visualization and Analysis of Crystal Structures. *J Appl Cryst* **2006**, *39* (3), 453–457. https://doi.org/10.1107/S002188980600731X.

(9) *CIF publishing tools*. https://publcif.iucr.org/services/tools/ (accessed 2025-03-24).

(10) Neese, F.; Wennmohs, F.; Becker, U.; Riplinger, C. The ORCA Quantum Chemistry Program Package. *The Journal of Chemical Physics* **2020**, *152* (22), 224108. https://doi.org/10.1063/5.0004608.

(11) Neese, F. Orca: An Ab Initio, DFT and Semiempirical Electronic Structure Package.

(12) San Diego Supercomputer Center (2025): Expanse. https://doi.org/10.1145/3437359.3465588.

(13) Perdew, J. P. Density-Functional Approximation for the Correlation Energy of the Inhomogeneous Electron Gas. *Phys. Rev. B* **1986**, *33* (12), 8822–8824. https://doi.org/10.1103/PhysRevB.33.8822.

(14) Lee, C.; Yang, W.; Parr, R. G. Development of the Colle-Salvetti Correlation-Energy Formula into a Functional of the Electron Density. *Phys. Rev. B* **1988**, *37* (2), 785–789. https://doi.org/10.1103/PhysRevB.37.785.

(15) Perdew, J. P. Erratum: Density-Functional Approximation for the Correlation Energy of the Inhomogeneous Electron Gas. *Phys. Rev. B* **1986**, *34* (10), 7406–7406. https://doi.org/10.1103/PhysRevB.34.7406.

(16) Neese, F.; Solomon, E. I. *Magnetism: From Molecules to Materials*; Wiley: New York, 2002; Vol. 4.

(17) Schaefer, A.; Horn, H.; Ahlrichs, R. TZVP. *J. Chem. Phys.* **1992**, *97*, 2571.

(18) Schäfer, A.; Huber, C.; Ahlrichs, R. Fully Optimized Contracted Gaussian Basis Sets of Triple Zeta Valence Quality for Atoms Li to Kr. *The Journal of Chemical Physics* **1994**, *100* (8), 5829–5835. https://doi.org/10.1063/1.467146.

(19) Weigend, F.; Ahlrichs, R. Balanced Basis Sets of Split Valence, Triple Zeta Valence and Quadruple Zeta Valence Quality for H to Rn: Design and Assessment of Accuracy. *Phys. Chem. Chem. Phys.* **2005**, *7* (18), 3297–3305. https://doi.org/10.1039/B508541A.

(20) Eichkorn, K.; Weigend, F.; Treutler, O.; Ahlrichs, R. Auxiliary Basis Sets for Main Row Atoms and Transition Metals and Their Use to Approximate Coulomb Potentials. *Theor Chem Acta* **1997**, *97* (1), 119–124. https://doi.org/10.1007/s002140050244.

(21) Eichkorn, K.; Treutler, O.; Öhm, H.; Häser, M.; Ahlrichs, R. Auxiliary Basis Sets to Approximate Coulomb Potentials. *Chemical Physics Letters* **1995**, *240* (4), 283–290. https://doi.org/10.1016/0009-2614(95)00621-A.
(22) Eichkorn, K.; Treutler, O.; Öhm, H.; Häser, M.; Ahlrichs, R. Auxiliary Basis Sets to Approximate Coulomb Potentials (Chem. Phys. Letters 240 (1995) 283-290). *Chemical Physics Letters* **1995**, *242*, 652–660. https://doi.org/10.1016/0009-2614(95)00838-U.
(23) Neese, F.; Wennmohs, F.; Hansen, A.; Becker, U. Efficient, Approximate and Parallel Hartree–Fock and Hybrid DFT Calculations. A 'Chain-of-Spheres' Algorithm for the Hartree–Fock Exchange. *Chemical Physics* **2009**, *356* (1), 98–109. https://doi.org/10.1016/j.chemphys.2008.10.036.
(24) Kossmann, S.; Neese, F. Comparison of Two Efficient Approximate Hartee–Fock Approaches. *Chemical Physics Letters* **2009**, *481* (4), 240–243. https://doi.org/10.1016/j.cplett.2009.09.073.
(25) Neese, F. An Improvement of the Resolution of the Identity Approximation for the Formation of the Coulomb Matrix. *Journal of Computational Chemistry* **2003**, *24* (14), 1740–1747. https://doi.org/10.1002/jcc.10318.
(26) Pettersen, E. F.; Goddard, T. D.; Huang, C. C.; Couch, G. S.; Greenblatt, D. M.; Meng, E. C.; Ferrin, T. E. Chimera. *J. Comput. Chem.* **2004**, *13*, 1605.
(27) Tulloch, A. A. D.; Danopoulos, A. A.; Winston, S.; Kleinhenz, S.; Eastham, G. N-Functionalised Heterocyclic Carbene Complexes of Silver. *J. Chem. Soc., Dalton Trans.* **2000**, No. 24, 4499–4506. https://doi.org/10.1039/B007504N.
